# Integrating the patient’s voice into the research agenda for treatment of chemosensory disorders

**DOI:** 10.1093/chemse/bjae020

**Published:** 2024-05-18

**Authors:** Claire Murphy, Pamela Dalton, Katie Boateng, Stephanie Hunter, Pamela Silberman, Jenifer Trachtman, Suz Schrandt, Bita Naimi, Emily Garvey, Paule V Joseph, Conner Frank, Abigail Albertazzi, Gurston Nyquist, Nancy E Rawson

**Affiliations:** Department of Psychology, San Diego State University, San Diego, CA, United States; Department of Psychiatry, University of California, San Diego, CA, United States; Monell Chemical Senses Center, Philadelphia, A, United States; The Smell and Taste Association of North America, Philadelphia, PA, United States; Monell Chemical Senses Center, Philadelphia, PA, United States; The Smell and Taste Association of North America, Philadelphia, PA, United States; Monell Chemical Senses Center, Philadelphia, PA, United States; ExPPect, LLC, Arlington, VA, United States; Department of Otolaryngology and Neurological Surgery, Thomas Jefferson University, Philadelphia, PA, United States; Department of Otolaryngology and Neurological Surgery, Thomas Jefferson University, Philadelphia, PA, United States; Division of Intramural Research, National Institute of Alcohol Abuse and Alcoholism, Section of Sensory Science and Metabolism and National Institute of Nursing Research, Bethesda, MD, United States; Department of Psychology, San Diego State University, San Diego, CA, United States; Department of Psychiatry, University of California, San Diego, CA, United States; Department of Psychology, San Diego State University, San Diego, CA, United States; Department of Otolaryngology and Neurological Surgery, Thomas Jefferson University, Philadelphia, PA, United States; Monell Chemical Senses Center, Philadelphia, PA, United States

**Keywords:** chemosensory disorders, smell, patient engagement, post-acute COVID, research agenda

## Abstract

World-wide some 658 million people were infected with coronavirus disease 2019 (COVID-19) and millions suffer from chemosensory impairment associated with long COVID. Current treatments for taste and smell disorders are limited. Involving patients has the potential to catalyze the dynamic exchange and development of new ideas and approaches to facilitate biomedical research and therapeutics. We assessed patients’ perceptions of the efficacy of treatments for chemosensory impairment using an online questionnaire completed by 5,815 people in the US Logistic regression determined variables predictive of reported treatment efficacy for patients aged 18 to 24, 25 to 39, 40 to 60, and 60+ yrs. who were treated with nasal steroids, oral steroids, zinc, nasal rinse, smell training, theophylline, platelet-rich plasma, and Omega 3. The most consistent predictor was age, with the majority of those 40 to 60 and 60+ reporting that nasal steroids, oral steroids, zinc, nasal rinse, and smell training were only slightly effective or not effective at all. Many of these treatment strategies target regeneration and immune response, processes compromised by age. Only those under 40 reported more than slight efficacy of steroids or smell training. Findings emphasize the need to include patients of all ages in clinical trials. Older adults with olfactory impairment are at increased risk for Alzheimer’s disease (AD). We speculate that olfactory impairment associated with long COVID introduces the potential for a significant rise in AD. Long COVID-associated chemosensory impairment increases the urgency for translational and clinical research on novel treatment strategies. Suggestions for high-priority areas for epidemiological, basic, and clinical research on chemosensory impairment follow.

## Introduction

Since the historic meeting of clinicians and researchers at the Monell Chemical Senses Center (Monell) in 2018 that resulted in the White Paper on treatments for chemosensory disorders published in Chemical Senses in May 2020 (Mainland et al. 2020), coronavirus disease 2019 (COVID) has dramatically changed the awareness and landscape of chemosensory dysfunction. The sheer number of people who have experienced chemosensory dysfunction has soared and the characteristics of the type of disorders that are most prominent, both in the acute and long-COVID phases, have evolved. Perhaps as important, awareness of chemosensory impairment has surged among clinicians, patients, and the general public.

An April 2022 patient engagement project was conducted by investigators from Monell, Jefferson Health, leadership of the Smell and Taste Association of North America (STANA), and consultants with patient engagement and chemosensory expertise. Information was gathered from patients with chemosensory impairment, most from long COVID, completed by 5,815 people in the United States and through listening sessions. These patient voices contributed much to our understanding of chemosensory disorders, their assessment, symptoms, diagnoses, treatment efficacy, and quality of life. These findings from the USA Smell and Taste Patient Survey (the Survey) will be used here to update the research agenda described in the White Paper.

Patients have participated in a number of critical surveys that have produced important data on the incidence and severity of chemosensory impairment from COVID (Green 2020; Parma et al. 2020; Gerkin et al. 2021; Weir et al. 2022). These studies heightened the urgency for research into the causes and treatment for COVID-related chemosensory impairment, research that will also benefit patients suffering from chemosensory impairment from other etiologies.

The patient voice has emerged as an important source of information about the impact of chemosensory impairment on quality of life (Nordin et al. 2011; Philpott et al. 2021). During the pandemic, a number of these studies investigated patient reports of chemosensory complaints and descriptions of symptoms. Information has also emerged regarding quality of life and patient perceptions of treatment efficacy.

Patient-centered outcomes research recognizes the need to incorporate the patient voice into research into treatments for chemosensory impairment. Patients express frustration with treatment strategies that produce little if any benefit, highlighting the need to maximize the potential for real gains in function from existing treatments by considering moderating variables. Patient voices increase the urgency for discovery research into the mechanisms that underlie chemosensory disorders and into new treatment strategies for chemosensory dysfunction. Patients express concern about medical provider’s background in chemosensory function and testing, suggesting the need for provider education programs, especially at the primary care level. Perhaps most importantly patients express a willingness to participate in research and in clinical trials. They are eager to have their voice incorporated into the effort to improve treatments, diagnosis, and reduce the negative impacts on their quality of life.

Thus, we here update the research agenda from the White Paper with key findings from the COVID pandemic and the emergence of a much stronger patient voice.

### Chemosensory disorders

Olfactory impairments include sensory loss, either complete (anosmia) or partial (hyposmia); distorted smell, either parosmia (distorted smell of odor that is present), or phantosmia (off or unpleasant smell when no odor is present). Age-related olfactory impairment is referred to as presbyosmia (see Table 1). Taste impairments include sensory loss, either complete (ageusia) or partial (hypogeusia), and distorted taste (dysgeusia). Chemesthetic impairments—altered trigeminal perception of temperature, touch, and pain—include altered nasal chemesthesis and altered oral chemesthesis (Green 2020). Olfaction, taste, and chemesthesis all contribute to the sensation of flavor. Patients often report taste impairment when flavor is altered because olfactory stimulation occurs retro-nasally during ingestion.

**Table 1.** Olfactory and taste dysfunction terms.

Olfaction	
Anosmia	Complete loss of smell
Hyposmia	Partial loss of smell
Parosmia	Distorted smell of an odor that is present
Phantosmia	Smell—often foul, off, or unpleasant—when no odor is present
Presbyosmia	Age-related olfactory loss

Taste	
Ageusia	Complete loss of taste
Hypogeusia	Partial loss of taste
Dysgeusia	Distorted taste

### Prevalence of olfactory impairment

Before the COVID pandemic, estimates of the incidence/prevalence of olfactory impairment in the United States ranged from 12% to 58%. Estimates from studies with objective testing were larger than those from self-report (Murphy et al. 2002; Hoffman et al. 2016; Schlosser et al. 2020; Desiato et al. 2021**).** The Beaver Dam study found that 24.5% of older adults had smell impairment, with prevalence increasing with age (Murphy et al. 2002). Prevalence was higher in men than women at every age sampled.

The most frequent causes of olfactory impairment in patients presenting to otorhinolaryngologists and to specialized clinics for Smell and Taste Disorders are inflammatory nasal sinus disease, head trauma, and postviral impairment; with a small percentage present with other etiologies; e.g. congenital anosmia (a rare condition), chemical burns, medications (Harris et al. 2006; Patel et al. 2022). A number of neurodegenerative diseases, particularly Alzheimer’s disease and Parkinson’s disease, are also characterized by olfactory impairment (Murphy et al. 1990; Graves et al. 1999; Woodward et al. 2017; Murphy 2019; Wheeler and Murphy 2021). In contrast to patients with nasal sinus disease, Alzheimer’s patients are largely unaware of their impairment (Nordin et al. 1995). Although they are unlikely to present to an otorhinolaryngologist with it as a symptom, some 85% of Alzheimer’s patients manifest olfactory impairment.

Since the advent of COVID, the numbers of individuals who have experienced smell impairment has soared. The degree and prevalence of smell impairment are dependent, peaking with the Delta variant. A systematic review indicated estimates of olfactory impairment during the acute phase range up to 77%, with objective testing revealing greater prevalence than subjective methods (Hannum et al. 2020). The majority of patients regain olfactory function after recovery from COVID (Hopkins et al. 2020; Lechien et al. 2020); however, recent data suggest that prolonged chemosensory impairment is associated with long COVID (Bussiere et al. 2022; Tan et al. 2022; Boscolo-Rizzo et al. 2023; Thaweethai et al. 2023). Psychophysical testing suggests that even after mildly symptomatic COVID a greater number of patients may suffer from prolonged olfactory impairment than self-report might indicate (Jensen et al. 2022). Acute symptoms include anosmia and hyposmia (Hannum et al. 2020), while prolonged symptoms include parosmia. It is currently estimated that more than 658 million people worldwide were infected with COVID and that loss or change in smell or taste is one of the most frequent symptoms associated with postacute sequelae of COVID infection, known as long COVID (Reese et al. 2023; Thaweethai et al. 2023).

### Treatments for chemosensory impairment

The most common etiologies of olfactory disorders are viral illness (COVID and non-COVID-related), chronic rhinosinusitis, allergic rhinitis, head trauma, aging, neurodegenerative disease, toxic exposure, medications, other diseases and conditions, and idiopathic (Harris et al. 2006; Patel et al. 2022). Current treatments for chemosensory impairment have had limited success (Harless and Liang 2016; Boesveldt et al. 2017; Doty 2019; Yan et al. 2019; Jafari and Holbrook 2022; Patel et al. 2022; Mahadev et al. 2023). More comprehensive information about individual treatment strategies appears in reviews by Hura et al. (2020); Jafari and Holcomb, (2022); Harless and Liang (2016); Helman et al. (2022); and Patel et al. (2022). Steroids, both nasal and oral, are frequently prescribed for inflammatory olfactory disorders (Patel et al. 2022). Smell training is one of the most frequently recommended treatments for olfactory dysfunction (Hura et al. 2020; Helman et al. 2022; Patel et al. 2022), though caution has been advised (Pieniak et al. 2022). Patients’ perceptions of how effective these treatments are and for whom is not clear. Studies have addressed efficacy, but there are limits to evidence-based outcome studies and study designs make interpretation of some results difficult. Many studies of efficacy have lacked control groups who complete a placebo arm (Patel et al. 2022). Because olfactory dysfunction shows spontaneous recovery, this complicates study interpretation. The average drop-out rate in olfactory training studies is high, e.g. 47% in a recent randomized clinical trial with 275 enrolled COVID patients (Khan et al. 2023). That study found no difference in UPSIT odor identification scores preintervention to postintervention between training and control groups. However, in studies that do report effects, drop-outs can inflate effect size because those who complete the study and are entered into the analysis are likely those for whom there is some efficacy.

### Patient engagement in evaluating treatment efficacy

Patient-centered research suggests the potential for incorporating information from the patient's voice into research into treatments for chemosensory impairment. Here we sought the patient’s voice to better understand the limitations and variability in efficacy of commonly prescribed treatments for olfactory disorders.

## Materials and methods

The USA Smell and Taste Patient Survey (See Supplementary Material) was made available online during two weeks from April 6, 2022 through April 28, 2022. A total of 6,100 participants provided electronic informed consent and completed this survey, of which 5,815 indicated they resided in the United States. A total of 5,528 indicated that they have smell and/or taste loss, referred to here as “patients.” We further cleaned the data for analysis by removing categories with underrepresented groups: we removed individuals less than 18 years old (*n* = 176), those who selected that their gender was nonbinary or unknown (*n* = 34), and those who did not know if their smell/taste dysfunction was due to COVID or not (*n* = 604), leaving us with a final sample size of 4877 patients included in this analysis (see Table 2). Before launching the survey in Qualtrics, all response quality options were enabled. This highlighted responses that may be bots and removed them from the data.

**Table 2.** Demographic information.

Demographic information	*N* (%)
Total	4,877
Gender	
Female	3,553 (72.85)
Male	1,324 (27.15)
Age	
18 to 24	571 (11.71)
25 to 39	1,450 (29.73)
40 to 60	1,887 (38.69)
60+	969 (19.87)
Race	
White	4,300 (88.17)
Black	273 (5.60)
Other	97 (1.99)
American Indian or Alaska Native	71 (1.46)
Prefer not to answer	64 (1.31)
Asian	52 (1.07)
Native Hawaiian or Pacific Islander	20 (0.41)
Ethnicity	
Not Hispanic or Latino	4,171 (85.52)
Hispanic or Latino	641 (13.14)
Prefer not to answer	65 (1.33)

Patients provided ratings of extremely effective, very effective, moderately effective, slightly effective, or not effective at all if they had been prescribed the following treatments: Nasal steroids, oral steroids, nasal rinse, smell training, theophylline, alpha lipoic acid, omega-3, platelet-rich plasma (PRP). Logistic regression analysis was used to investigate significant differences between those who rated treatments as not effective or slightly effective versus those who rated them moderately effective, very effective, or extremely effective. COVID etiology, age, gender, documented disorder, and disorder length were entered into the model as predictors of efficacy. The Wald Test was used to assess the significance of individual coefficients, followed by pairwise Wald’s tests with Tukey’s adjustments for multiple comparisons when appropriate. Listening sessions followed with small groups. All analyses were done in R Studio (Version 2022.12.0 + 353).

The study complies with the Declaration of Helsinki for Medical Research involving Human Subjects. The protocol was reviewed by the University of Pennsylvania Institutional Review Board and determined to meet the criteria for IRB review exemption. Participants provided written informed consent.

## Results

### Demographics

Survey participants were 73% female, and 59% over age 40 (see Table 2 for demographics). A total of 74% of patients reported having a smell or taste disorder because of COVID and 79% reported ongoing dysfunction. In those with ongoing dysfunction, the average duration was 3.65 years. For those who recovered, the average duration of smell/taste dysfunction was 1.47 years. We do not have complete information on the duration of steroid use or olfactory training. As occurs in practice, different patients employed different olfactory training regimens and complied for different durations. 55% of participants consulted a healthcare provider because of their smell or taste disorder symptoms. Of those, 64% reported consulting a family practitioner, 37% an otolaryngologist, 5% a Taste and Smell Clinic, and less than 10% other providers. Those consulting an otolaryngologist were more likely to receive a diagnosis. The best predictor of receiving a diagnosis was otolaryngologist evaluation, followed by smell testing. Those who received smell testing had 1.83 higher odds of receiving a diagnosis (Naimi et al. 2023).

Of those surveyed, 1,890 reported using treatments for chemosensory dysfunction. Of these, the most used were nasal steroids (43%) and smell training (31%). The most consistent predictor of efficacy was age with the majority of those 40 to 60 and 60 + rating nasal and oral steroids, zinc, nasal rinse, and smell training only slightly effective or not effective at all. Differences were not significant for theophylline or PRP but samples were small.

**Fig. 1.**
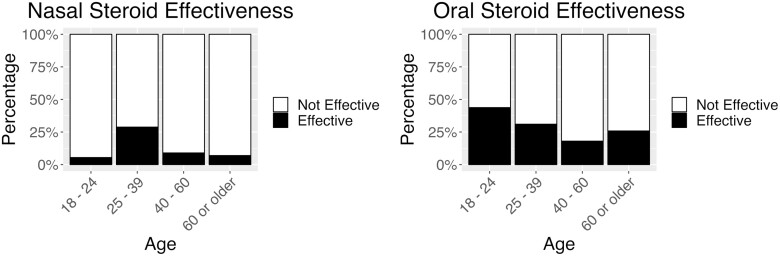
Nasal (left) and oral (right) steroid effectiveness by age group. The black bar indicates the percentage of participants in each age group who found steroids moderately, very or extremely effective, and the white bar indicates the percentage who found the steroids slightly effective or not effective at all.

**Fig. 2.**
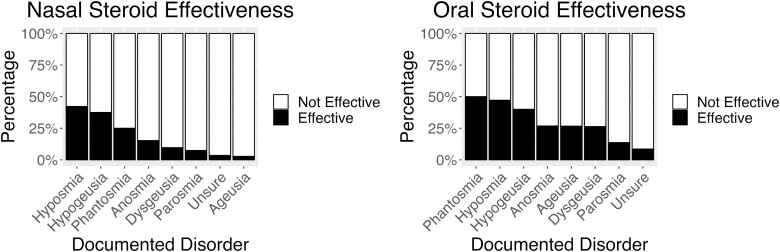
Nasal (left) and oral (right) steroid effectiveness by documented disorder. For each disorder the black bar indicates the percentage of participants in each group who found steroids moderately, very or extremely effective, and the white bar indicates the percentage who found the steroids slightly effective or not effective at all.

Nasal steroids were the most frequently used treatment by these patients. Age (*X*^2^ = 33.9, df = 3, *P* < 0.001) and documented disorder (*X*^2^ = 27.8, df = 7, *P* < 0.001) influenced patient rated efficacy of nasal steroids. Those 25 to 39 years rated nasal steroids more effective than those 40 to 60 (*P* = 0.002) and 60 + (*P* < 0.001). Nasal steroids were rated slightly effective or not effective at all by 91% of those 40 to 60 years and 93% of those 60 + years (see Fig. 1). Those with parosmia (7%) rated nasal steroids less effective than those with hyposmia (42%; *P* = 0.025) and hypogeusia (38%; p = 0.043) (see Fig. 2). Furthermore, males found nasal steroids more effective than females (OR 1.85, 95% CI [1.03, 3.33], *P* = 0.039).

Age was also a significant predictor of the efficacy of oral steroids (*X*^2^ = 13.4, df = 3, *P* < 0.004). Those 18 to 24 rated oral steroids more effective than those 40 to 60 (*P* = 0.002). Those 25 to 39 rated oral steroids more effective than those 40 to 60 (*P* = 0.032) and 60 + (*P* = 0.04). Although oral steroids were rated higher than nasal steroids, the majority of those 40 to 60 and 60 + rated both nasal and oral steroids as only slightly effective or not effective at all (see Fig. 1).

**Fig. 3.**
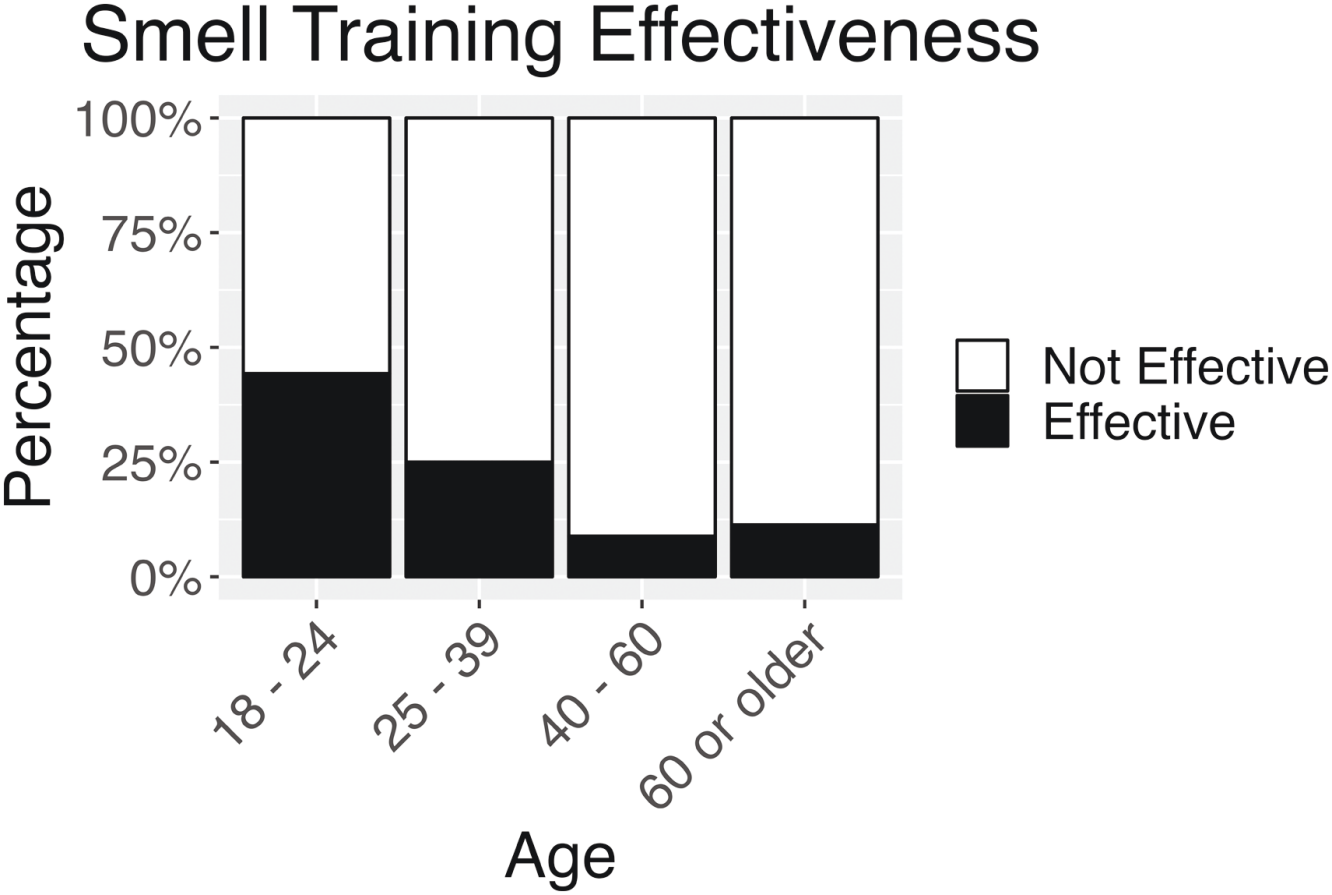
Rated smell training effectiveness was significantly different by age group. The black bar indicates the percentage of participants in each group who found smell training moderately, very or extremely effective, and the white bar indicates the percentage who found smell training slightly effective or not effective at all.

What did the patient-centered survey research reveal about smell training? We investigated rated efficacy in those 790 individuals who reported using smell training. Logistic regression with COVID etiology, age, gender, and documented disorder in the model revealed significant differences between those who rated smell training as not effective or slightly effective versus those who rated smell training as moderately effective, very effective, or extremely effective revealed age, gender and documented disorder to influence efficacy. Patients’ age (*X*^2^ = 27.5, df = 3, *P* < 0.001) significantly influenced efficacy. Those 18 to 24 rated smell training more effective than those 25 to 39 (*P* = 0.032), 40 to 60 (*P* < 0.001), and 60 + years (*P* < 0.001). Those 25 to 39 rated smell training more effective than those 40 to 60 (*P* = 0.009) and 60 + years (*P* = 0.048). Smell training was rated slightly effective or not effective at all by 89% of those 60 + and 91% of those 40 to 60 years (see Fig. 3).

**Fig. 4.**
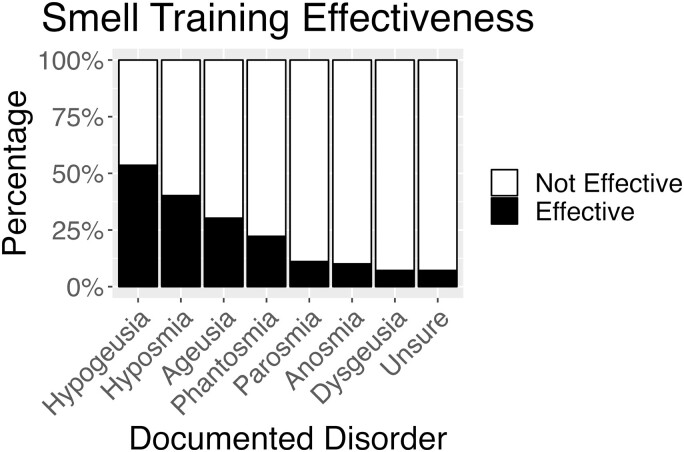
Reported smell training effectiveness as a function of chemosensory disorder. For each disorder, the black bar indicates the percentage of participants in each group who found smell training moderately, very or extremely effective, and the white bar indicates the percentage who found smell training slightly effective or not effective.

Efficacy of smell training was also driven by chemosensory disorder (*X*^2^ = 38.2, df = 7, *P* < 0.001) (see Fig. 4). Those with anosmia rated smell training less effective than those with hyposmia (*P* = 0.006) and hypogeusia (*P* < 0.001). Those with hypogeusia rated smell training more effective than those with parosmia (*P* = 0.007) and dysgeusia (*P* = 0.009). Those who were unsure of their diagnosis rated smell training less effective than those with hyposmia (*P* = 0.025) or hypogeusia (*P* = 0.002). The effects on hypogeusia (diminished taste) might suggest the effects of training on nonsensory specific aspects of task performance, e.g. attention, concentration; or the tendency to equate flavor (taste + smell) with taste when describing symptoms of loss. Finally, females rated smell training less effective (12%) than males (34%; OR = 1.90, 95% CI [1.12, 3.20], *P* = 0.016).

### Listening to the patient voice

In listening sessions that followed with smaller groups, patients expressed the need to know the cause of their disorders and whether there was evidence that a particular treatment was effective. They expressed interest in smell assessment at the annual physical with their primary care physician and excitement about participating in clinical trials. They were also frustrated with treatment strategies that produce little if any benefit, suggesting the need to maximize the potential for real gains in function by considering moderating variables and new treatment strategies (see Table 3).

**Table 3.** Patient voices.

Patients expressed a need to know the causes of their disorders and whether there is evidence that smell training is efficacious. They were enthusiastic about smell assessment at the annual physical with their primary care physicians and in participating in clinical trials.
“I know for a lot of us, **we don’t really know what the cause is**, whether it’s a problem with one’s nerves not working, nerves being dead, nerves being disconnected or something else. I think **it’d be helpful to know**.”
“**wouldn’t be nice if in your annual physical your GP would ask, especially as you get older, “And how’s your sense of smell?”**
“Dr. ___ had me do all the smells. I had I don’t know how many, I think eight bottles of different oils. And **I faithfully sniffed them twice a day for about two months. And nothing ever happened and never smelled another thing. Or tasted anything. Ever.”**
“**whether the smell training** that he has been doing has, if there’s any proof that it actually does something or it actually works.”
**“And I can answer the question about why people drop out.** I couldn’t smell anything. I did smell training religiously for two months and really couldn’t differentiate between one and the other scent because pretty much nothing came through”
“I’ve seen **probably half dozen doctors** of various sorts to this over the years
“**It’s talked about a lot more now by friends of mine.** Specific friends are moaning that they have lost their sense of smell because of COVID. And so, I haven’t had a sense of smell since 2004. And most of their sense of smell has come back. But **a lot of people that I know have lost sense of smell and taste because of COVID. So, there’s a lot more knowledge out there”**
“I did meet a doctor last year who’s from UCLA that said because of COVID there’s a **tremendous amount of interest in smell loss**. And he said he’s working on something that regenerates neurons. **And I’m really excited about it.** And he told me he’s going to tell me when it goes into clinical trials so I can **see if I can get in that clinical trial.”**

## Discussion

Patient-centered research utilizing the USA Smell and Taste Patient Survey supports the benefit of incorporating the patient voice into research into treatments for chemosensory impairment. The results revealed the importance of considering the age range for which treatments are effective and the tailoring of treatment to the underlying olfactory symptoms. Treatment efficacy was rated higher in (i) those 25 to 39 years old and (ii) in those with hyposmia rather than anosmia. The most consistent finding was the difference in efficacy between younger and older adults, with significantly reduced efficacy in those over 40 years. This has important implications for the rising use of precision medicine and for clinical trials that consider the critical factors that modify treatment effectiveness. The results emphasize the importance of investigating the underlying mechanisms for treatment efficacy in anosmia, hyposmia, and parosmia. Most strikingly, they emphasize the need for research on the underlying mechanisms for chemosensory impairment and the development of more effective treatments for chemosensory disorders.

The results resonate with a number of recent reviews of the literature on current strategies for chemosensory impairment. Many current treatment strategies lack the essential support of evidence-based, randomized, clinical trials with appropriate control groups, conducted over the lifespan. The Oxford Center for Evidence-Based Medicine Criteria 2011 define the criteria for studies with level 1 evidence as a properly powered and conducted randomized clinical trial or systematic review, and for level 2 as a well-designed controlled trial that lacks randomization or a prospective comparative cohort trial.

A systematic review by Hura et al. (2020) of existing clinical trials of treatment for postviral olfactory dysfunction identified 36 studies that investigated the effects of medical, surgical, or olfactory training interventions on greater than 5 subjects. These included 7 studies with level 1 (properly powered and properly conducted randomized clinical trial, systematic review) and 4 with level 2 (well-designed controlled trial without randomization; prospective comparative cohort trial), and 25 studies with lower levels of evidence-based medicine. Overall, the review revealed heterogeneous studies with insufficient high-level evidence limiting recommendations for most treatments. Topical or local steroids were offered as an option with a balance of risk versus benefit. Olfactory training was recommended. The most striking finding was the need for evidence-based investigations of treatments for postviral olfactory dysfunction.

Helman et al. (2022) conducted a systematic review of 12 treatments and 2 interventions for postviral olfactory dysfunction (PVOD). Thirteen studies that evaluated the efficacy of medical therapies for the treatment of PVOD were identified for review. Topical steroids and caroverine and alpha-lipoic acid were supported as possible beneficial interventions for postviral olfactory disorders; minocycline, vitamin A, and zinc sulfate were not supported. The low risk/benefit ratio of topical steroids was suggested as support for its recommendation as a first-line option targeting inflammation. Olfactory training was suggested to be supported; however, spontaneous recovery was acknowledged as a limitation of the assessment of its efficacy. Helman et al concluded that although data to date suggest these treatments are likely to hasten recovery over spontaneous recovery, in order to substantiate these treatments, more research is required.

Not evaluated in that review or in a number of other studies was the problem with compliance with many weeks of olfactory training and the issue that dropouts pose for interpretation of analyses that include only those who complete a study. The low risk/benefit ratio, low cost, and absence of complications were considered supportive of its use, though patient input regarding the toll of the training, particularly for those who experience little benefit, was not included. As with a number of reviews, the effect of patient age was not addressed. Strategies that target inflammation or regeneration are highly likely to be impacted by patient age. Regeneration of olfactory epithelium has been suggested as a mechanism for smell training (Pieniak et al. 2022) and age-related neurogenic exhaustion as a mechanism for presbyosmia, or age-related olfactory loss (Child et al. 2018).

In an exhaustive position paper reviewing the available evidence for treatments for olfactory dysfunction, Patel et al (2022) found level 1 or 2 evidence to support recommendations for only a few of the existing treatment strategies currently used to directly treat olfactory dysfunction. Treatments recommended for anosmia or hyposmia due to specific etiologies included omega-3 for olfactory dysfunction after endoscopic skull base surgery and referral to a specialist for treatment of chronic rhinosinusitis. Topical steroids and olfactory training were recommended after postviral infection or idiopathic causes, with oral zinc and topical vitamin A as options and topical sodium citrate only for short-term improvement. Topical steroid irrigations and olfactory training were also recommended after trauma, with oral zinc and topical vitamin A as other options. There was insufficient data to provide recommendations for alpha lipoic acid, toki-shakuyakusan, intranasal theophylline, intranasal insulin, PRP, oral steroid, and nasal steroid sprays as efficacious treatments for olfactory dysfunction. Overall there was low evidence for treatments for phantosmia/parosmia with no recommendations and only suggestions for options.

Assessment of the response to steroids is complicated by the fact that many patients who respond to steroids may have had a residual sinusitis, present in some 5% to 12% of the population (Dietz de Loos et al. 2019). Reduction in the accompanying resident inflammation may be responsible for the response to steroids. Further, the response is difficult to explain by steroids reaching the olfactory cleft because nasal sprays or drops do not reach the olfactory cleft with a standard administration (Benninger et al. 2004).

There were specific recommendations against systemic vitamin A, minocycline, systemic phosphodiesterase inhibitors (PDEIs; theophylline, pentoxyphylline, caffeine), and for intranasal zinc specifically for nonpost traumatic olfactory dysfunction. Numerous studies have concluded that results from treatment with zinc are no better than spontaneous recovery (Patel et al. 2022). Intranasal zinc has been associated with anosmia in humans (Alexander and Davidson 2006). Research in mice demonstrated that intranasal zinc sulfate caused temporary but essentially total disruption of functional connections from the olfactory epithelium to the bulb (McBride et al. 2003).

Research to evaluate and optimize current treatment approaches is needed. Level 1 evidence is available to inform treatment with only a small number of existing approaches though a number of other treatments show some promise and should be subjected to evidence-based research evaluation. Randomized, clinical trials with appropriate comparison groups are urgently needed for these potential treatments as well as for novel treatments used to combat COVID.

Although loss or change in smell is one of the most frequent symptoms associated with long COVID (Reese et al. 2023; Thaweethai et al. 2023), given the variability over time of guidelines for reporting COVID infections and symptoms, including olfactory dysfunction, an estimate of the number of COVID patients recovering olfactory function is difficult to ascertain. Estimates of the number of patients with long COVID-related olfactory dysfunction differ for studies of clinical patients and those recruited from the community, differ for self-report and objective testing, and differ across variants and over time (Bussiere et al. 2022; Prem et al. 2022; Tan et al. 2022; Boscolo-Rizzo et al. 2023; Thaweethai et al. 2023). Recent estimates range broadly: 27.9% in the Boscolo-Rizzo et al. (2023) study of consecutively assessed hospital patients considered suitable for home management, 51% among healthcare workers 4-7 months after infection in 2020 (Bussierre et al. 2022), 76.5% in the Prem et al. (2022) study that recruited patients with proven COVID infection and subjective olfactory dysfunction who contacted the study for inclusion. In the current study 79% of participants who reported having had COVID, a self-selected group, had ongoing smell/taste dysfunction. Different recruitment strategies yield different, valuable information.

Among the respondents to the current survey, older adults reported significantly poorer efficacy of current treatments. A number of factors associated with increasing age in the olfactory system may affect response to treatment. The peripheral olfactory system undergoes significant changes: The olfactory epithelium is thinner, contains fewer olfactory receptor neurons, has less mucus, and is hypothesized to be associated with increasing atrophy and regenerative exhaustion. As the regenerative capacity of the epithelium is reduced, patches of the olfactory epithelium are replaced by respiratory-like epithelium (Loo et al. 1996; Schwob et al. 2017; Child et al. 2018). Recent research from Goldstein’s group, suggesting a role for cytokines and TP63 - a transcription factor that acts to prevent stem cell differentiation—in disrupting neurogenesis in presbyosmia patients over 65, may represent both a model for olfactory loss and the potential for a therapeutic target (Oliva et al. 2022). Olfactory receptor cell response is less specific in older adults (Rawson et al. 2012). Both anterograde and retrograde degeneration may impact the olfactory system as central targets undergo age-associated atrophy. Entorhinal cortex thickness decreases with age (Fjell et al. 2014). Event-related potential studies show slower processing of olfactory stimuli with aging (Murphy et al. 2000; Stuck et al. 2006; Morgan and Murphy 2010); and functional MRI techniques show significantly reduced activation to odor stimulation in olfactory processing areas; i.e. amygdala, piriform cortex, orbital frontal cortex, and enthorhinal cortex (Cerf-Ducastel and Murphy 2003; Wang et al. 2005).

Interestingly, in pre-COVID assessments of patients presenting to chemosensory clinics, a number of papers reported greater numbers of female than male patients with olfactory loss secondary to upper respiratory viral infection (Goodspeed et al. 1987; Deems et al. 1991; Temmel et al. 2002; Harris et al. 2006). Although they did not assess the efficacy of various treatment strategies by gender, Prem et al. (2022), reported that more women than men reported long-lasting olfactory dysfunction after COVID. That study recruited patients with proven COVID infection and subjective olfactory dysfunction who contacted the study for inclusion. They proposed that “more women than men are interested in individual olfactory function and improvement of its dysfunction.” In the current study, ratings of treatment efficacy were lower for women than for men. This may be related to their ratings of their sense of smell prior to the onset of symptoms. The percentage reporting that their sense of smell had been “somewhat good” or “extremely good” was 87.55% in females and 58.47% in males. One might speculate that individuals with better olfactory function may be more likely to notice a deficit and to be more sensitive to the degree of loss and functional recovery.

Finally, to advance new treatment approaches, research into the underlying mechanisms for different etiologies of olfactory impairment is critically needed. A living systematic review of the benefits and harms of treatments for persistent COVID-related olfactory dysfunction has been established as a Cochrane Database Systematic Review (Webster et al. 2022).

## Conclusion

It is important to recognize that the data from the USA Smell and Taste Patient Survey reflect the participant pool: Those who responded to an online survey of chemosensory impairment. Nevertheless, the findings suggest the importance of future research in large-scale studies and in randomized clinical trials that investigate patients who are treated for chemosensory impairment. They suggest the need to differentiate among effects on anosmia, hyposmia, and parosmia, to understand the mechanisms underlying the different etiologies for olfactory impairment, and to analyze effects on patients of different age groups and include control groups to assess spontaneous recovery and address drop-outs.

Patient-centered research supports the benefit of incorporating the patient voice with research into treatments for chemosensory impairment. As evidenced by the Survey and listening session results, patients shared feedback on a wide range of topics impacting their lived experience with chemosensory dysfunction. There are gaps in research, resources, training, and education that must be addressed to better serve this growing patient population and integrate their voice throughout the research endeavor. The findings suggest a number of next steps for a future research agenda to determine who will benefit from treatment.

### Next steps in patient-centered research

#### Develop partnerships with patient groups, clinicians, and basic scientists to facilitate patient-centered research, discovery, and therapeutics

This patient-centered engagement project suggests the heightened need to incorporate the patient voice into research into treatments for chemosensory impairment, from current high-profile chemosensory disorders affecting large numbers, as in long COVID, to rare diseases like congenital anosmia. Patients express concern about medical provider’s background in chemosensory function and testing, suggesting the need for input to medical provider education and training programs. Patients expressed frustration with methods that produce little if any benefit, suggesting the need to maximize the potential for real gains in function from existing treatments and discovery research into new treatment strategies. Perhaps most importantly patients express a willingness to participate in research—many are eager to participate in clinical trials (see Table 3).

The White paper of 2020 (Mainland et al. 2020) wisely suggested the formation of patient advocacy groups to

1) Contribute to public awareness and elicit financial support;
2) Provide insights into the needs of people living with chemosensory disorders;
3) Serve on scientific advisory boards;
4) Participate in scientific workshops and give interviews and presentations about their experiences with chemosensory disorders;
5) Organize registries of patients for research participation;
6) Advise on how to communicate with other patients;
7) Work with clinicians to establish insurance reimbursement for care for chemosensory dysfunction.

The current assessment reaffirms the need for patient advocacy groups. Physicians, scientists, and clinicians can help patient advocacy groups to be their most effective by collaborating with patients, and sharing their expertise in scientific methods and techniques, while patients can provide valuable insight into the patient experience, which can help guide research priorities and study design.

Patients can participate in studies, and clinical trials and join patient registries to gain opportunities to participate in current and future research. Patients can advocate for policy change. For example, they can advocate for universal chemosensory testing, increased funding for research into rare diseases (like congenital anosmia), or improved access to healthcare services for underserved populations. Real progress has been made in the formation and development of patient advocacy groups, such as STANA: the Smell and Taste Association of North America, CKOS (Chrissi Kelly on Smell, formerly Abscent), Association Anomie.org, Fifth Sense, reuksmaakstoornis, and the Patient Advisory Group for Chronic Rhinosinusitis (see Table 4).

**Table 4.** Resources and patient organizations.

Organization	Website	Email
Abscent(See CKOS)	https://abscent.org.uk	
Asociacion Española de la Anosmia	https://asociacionanosmia.com/	
Association Anosmie	https://www.Anosmie.org	
Association for Chemoreception Sciences	http://www.achems.org	info@achems.org
CKOS Chrissi Kelly on Smell	https://www.chrissikelly.com/	
Fifth Sense	https://fifthsense.org.uk	info@fifthsense.org.uk
Monell Chemical Senses Center	http://monell.org	FCOI@monell.org
National Institute of Deafness and Other Communicable Disorders (NIDCD)	http://www.nidcd.nih.gov	nidcdinfo@nidcd.nih.gov
Reuksmaakstoornis	http://www.Reuksmaakstoornis.nl	secretariaat@reuksmaakstoornis.nl
Smell and Taste Association of North America (STANA)	https://thestana.org	info@thestana.org
Swirl of Hope	https://www.covidswirl.com/	info@covidswirl.com
World Taste and Smell Association	https://www.tasteandsmell.world/	

Patients have shown that they can not only be advocates, but they can be true partners in research teams with clinicians and basic scientists to facilitate patient-centered investigation. The Survey is one clear example of the importance of patient voices. Representatives of STANA, the Smell and Taste Association of North America, themselves anosmic, contributed to issues to be addressed, to the development of the questions for the survey, to disseminating the survey, to interpreting the findings, to disseminating the findings here and at the Association for Chemoreception Sciences 2023 Annual Meeting (Dalton et al. 2023; Garvey et al. 2023; Murphy et al. 2023; Naimi et al. 2023; Rawson et al. 2023), to use the findings to update medical providers at a Resident Education teaching session, and members of AChemS in a unique Webinar (June 9, 2023). As the field moves forward, patient voices have the potential to be powerful agents in research, discovery, and the development and funding of therapeutics.

#### Develop chemosensory testing strategies that can be used for universal smell testing for population screening and by primary care providers and engage in high-level evidence-based studies to demonstrate utility to provide diagnostic value and facilitate reimbursement for testing

The COVID pandemic has made clear the need for chemosensory testing techniques that can be used at the primary care level (Saraswathula et al. 2023). In many studies at the population level, olfactory testing has relied on odor identification tests for identifying dysfunction. Current tests are also useful for identifying patients unable to detect and name suprathreshold odors but they are under-utilized and physicians are not generally reimbursed for their use in the United States (Saraswathula et al. 2023). Further development of rapidly administered, inexpensive, user-friendly tests that can be immediately interpreted is needed. The field needs tests that require little or no examiner time, data acquisition through user-friendly applications, and machine learning to produce algorithms for scoring that can increase potential for adoption by clinicians, for surveillance testing, and for use in large-scale epidemiological studies. These advances will be important to assess chemosensory impairment associated with long COVID and to tailor treatment to impairment. Hyposmia and parosmia are symptoms of long COVID. Tests that target these symptoms directly are needed to provide information that identifies and follows olfactory impairment in patients with long COVID, in addition to existing, more general odor identification tests for assessment of olfactory dysfunction associated with acute COVID. Assessment of odor identification will remain important as tests are developed for following the long-term effects of COVID and for its potential effects on the development of neurodegenerative diseases that compromise olfactory function. In response to COVID, the NIH has invested in projects to accelerate the development of chemosensory testing through its Rapid Acceleration of Diagnostics initiative. These tests will be important for assessment of the effects of new COVID variants and other viral diseases in the future.

Research is needed to establish the sensitivity and specificity of new and existing tests that target specific aspects of chemosensory function or specific conditions. Research to support the efficacy of testing will facilitate the process of approval of reimbursement codes for testing, a critical step that is increasingly recognized as important to reduce inequities in the diagnosis and treatment of olfactory dysfunction as improved evidence-based interventions become available (Saraswathula et al. 2023). Patient-centered outcome research indicates the need not only for patient education about chemosensory testing but for medical provider education to increase its implementation. The patients’ voices advocating for the availability of testing will be important at all levels of this effort: in research, education, and policy.

#### Use biomedical data science to investigate chemosensory disorders through electronic health records, epidemiological studies, and other large data sets

It is critical to understand the underlying etiologies of olfactory dysfunction to develop better-targeted therapies. Electronic medical records have the potential to yield information on patient characteristics associated with different etiologies and further chart the trajectories of recovery using different treatment strategies. Harmonization of data from electronic records and the use of standardized, normed, and highly reliable chemosensory tests in different research and health care settings would facilitate research using data-driven discovery methods.

In addition to medical records, existing data on taste and olfaction in large cohort studies represent a rich source of information that can inform chemosensory dysfunction and the effectiveness of treatment approaches. Studies such as NHANES; the Beaver Dam study; the Health and Retirement Study; Health, Aging and Body Composition Study (Health ABC), incorporated olfactory and/or taste testing within the context of studies with a host of health variables, some including assessments of other sensory systems. A number of large, well-characterized cohort studies have more recently incorporated olfactory and/or taste testing and data will be available for future research, e.g. the VETSA study of veterans; SOL-INCA, the Study of Latinos—Investigation of Neurocognitive Aging. Including olfactory functional assessment in ongoing longitudinal studies such as NHANES is critically important. Because of time constraints and participant burden, epidemiological and prevalence studies often demand rapidly administered tests that can be given and interpreted by nonexperts. This will be particularly important for longitudinal investigation into the long-term consequences of long-COVID on chemosensory impairment and emphasizes the need for such tests to be available. Such tests may be particularly useful in assessing children, about whom very little data are available on the effects of COVID or long COVID, yet the influence of reduced olfactory function on food selection and preference during this sensitive period in development may be especially important. As additional studies include chemosensory testing or additional waves of testing occur, incorporating the patient's voice into the questions that accompany testing has the potential to increase insight into the data collected. The importance of developing data that represent diverse cohorts cannot be over-emphasized.

#### Subject treatment strategies to randomized, clinical trials with control groups and patients across the lifespan

The Oxford Center for Evidence-Based Medicine Criteria define the criteria for studies with level 1 a properly powered and conducted randomized clinical trial or systematic review, and level 2 well-designed controlled trial that lacks randomization or a prospective comparative cohort trial.

Many current treatment strategies for olfactory dysfunction lack the essential support of evidence-based, randomized, clinical trials with appropriate control groups as defined by the 2011 Oxford Center for Evidence-Based Medicine Criteria. Level 1 evidence is available to inform treatment with only a small number of existing approaches though a number of other treatments show some promise and further studies are warranted (Hura et al. 2020; Helman et al. 2022; Jafari and Holbrook 2022; Patel et al. 2022). Randomized, clinical trials with appropriate comparison groups are urgently needed as new treatments for chemosensory disorders are considered, such as platelet-rich plasma and stellate ganglion blocks. As treatments used to combat COVID and other treatments for systemic disease associated with olfactory disorders are assessed, it will be critical to subject these to the highest levels of evidence-based medical research. Patient-based research also raises the questions of patient compliance and patient satisfaction with the degree of improvement from treatments. Incorporating the patient's voice into future research into these issues will improve outcomes.

#### Build on lessons learned from COVID

The emergence of olfactory and taste disorders as symptoms of COVID and the research that then characterized the types of chemosensory disorders that patients reported set the stage for investigations by physicians and basic scientists into the type of disorders caused by COVID, and indeed by different variants. Early reports of chemosensory impairment from COVID, both those that relied heavily on self-report and those that included some sensory testing, suggested that olfaction was most vulnerable to the virus, though patients reported both taste and chemesthetic symptoms (Green 2020; Hannum et al. 2020; Iravani, Arshamian, Ravis et al. 2020; Parma et al. 2020; Gerkin et al. 2021). Importantly, hyposmia was more prevalent in the acute phase, parosmia became a later focus (Green 2020; Hannum et al. 2020; Hopkins et al. 2020; Parma et al. 2020; Pellegrino et al. 2020; Tong et al. 2020; Gerkin et al. 2021; Gary et al. 2023). A sense of urgency to understand the effects of COVID on patients’ chemosensory systems gripped physicians and basic scientists alike as the number of patients with COVID-related dysfunction grew. As the pandemic wore on, long COVID-associated chemosensory impairment emerged as an important etiology. This patient-first approach was key to the rapid progress made in understanding the acute effects of COVID on olfaction and is now informing investigation into the effects of long COVID. This information informed and spurred basic research into the mechanisms of COVID-related olfactory system dysfunction.

Early work on human biopsy tissue from the olfactory epithelium established that COVID directly targets sustentacular cells and olfactory stem cells (Brann et al. 2020). Using single-cell RNA-sequencing and immunohistochemistry, Goldstein’s group later reported fewer olfactory receptors, particularly mature OMP + neurons, relative to sustentacular cells and sustained inflammatory signaling in sustentacular cells in the olfactory epithelium of a small sample of hyposmics who had long COVID. Persistent infiltration of T cells in the epithelium after the virus is no longer detected may suggest a selective immunological response to previous infection that differs from the acute response and may represent a potential mechanism for olfactory impairment in long COVID. Further studies using standardized biopsy techniques, larger sample sizes, with longer follow-ups are warranted (Finlay et al. 2022). The race to understand and treat COVID pushed the envelope and new discoveries surged around olfactory receptor cell and sustentacular cell function. Recent and emerging research has focused attention on inflammatory markers and the interaction of inflammation with regenerative processes in the olfactory epithelium (Chen et al. 2019). Depression of regeneration in the face of inflammation is a demonstrated response to viral infection. Research into this interaction needs to include the moderating effects of critical variables such as age, prior viral insults, or toxic exposures on regeneration. How this interacts with the inflammatory response is unknown. Lessons learned from COVID increase the urgency of research into regeneration in the face of inflammation.

Investigation of the mechanisms underlying postviral olfactory dysfunction from COVID in hamsters revealed the importance of animal models (Zhang et al. 2021). Studies of COVID patients consistently have reported that older adults are more at risk for disease and when infected suffer from more severe disease, more often resulting in hospitalization or death (O’Driscoll et al. 2021). Response to treatment for chemosensory impairment from COVID shows a similar pattern, suggesting the need for a precision medicine approach (Liu et al. 2022). Research into the causes of chemosensory impairment from COVID has revealed important information, however, research on the mechanisms underlying acute and, particularly, long COVID is critical to develop treatment strategies to prevent, halt, and reverse chemosensory impairment from COVID and future viral illnesses that may substantially increase the prevalence of chemosensory dysfunction.

#### New approaches to assess chemosensory system disorders at the periphery

The White paper of Mainland et al. (2020) offered 7 suggestions for high-yield next steps to result in a more vigorous approach to the development of treatments for chemosensory disorders. First among the priorities was supporting new avenues of research into cellular approaches. Those avenues centered first around increasing knowledge of the regeneration of taste and olfactory receptor cells, with accompanying knowledge of supporting cells, progenitor cells, and other key factors. We renew the call for research into the development, degeneration, and regeneration of taste and olfactory tissues at the cellular level, COVID accelerated investigation into viral insult to the receptor cells, the sustentacular cells, and the basal cells of the olfactory epithelium. Research examining tissue from the olfactory epithelium of patients with hyposmia yielded provocative information about immune cell infiltration and altered gene expression in long COVID-associated olfactory impairment (Finlay et al. 2022). Continued commitment to discovery needs to be invested in assessing the peripheral olfactory system for chemosensory impairment associated with this and other causes.

#### Treatment strategies for central impairment

Current treatment strategies for olfactory dysfunction largely target peripheral causes; however, there is accumulating evidence that long COVID may involve damage to central structures that process olfactory information. Douaud et al. (2022) study used structural MRI to study the effects on the brain in COVID patients from the UK Biobank (Sudlow et al. 2015) and found significant loss of volume in orbitofrontal cortex and parahippocampal gyrus, areas that process olfactory information, and greater changes in markers of tissue damage in areas connected to the primary olfactory cortex.

To better assess central chemosensory function in long-COVID and other disorders that exhibit chemosensory impairment; neuroimaging, noninvasive recordings, and other techniques to assess the integrity and function of central olfactory processing areas would need to be evaluated and optimized for clinical assessment. Noninvasive recording of signals from the human olfactory bulb has recently been reported by Lundström’s group (Iravani, Arshamian, Ohla et al. 2020). They obtained signals from EEG electrodes at the nasal bridge that represent responses from the bulb—recordings they term Electrobulbogram (EBG). Programs such as NeuroQuantTM yield interpretable values for hippocampus and hippocampal areas based on MRI, metrics useful for research, clinical trials, and to the neurologist diagnosing neurodegenerative disease (Yu et al. 2014). Neuroimaging that yields a normative score for the integrity of key regions for chemosensory processing in the brain would optimize its use in research, clinical trials, and the physician’s office.

Recent reports of reduced olfactory bulb volume on MRI in COVID patients raise the possibility of using structural imaging to assess olfactory bulb integrity in olfactory disorders (Fournel et al. 2020; Kandermirli et al. 2021; Frosolini et al. 2022). Standardized, normative data would need to be developed if this approach proves to be reliable and valid. Because of susceptibility artifacts near the sinuses, functional neuroimaging of the olfactory bulb with 1.5 and 3T scanners has been difficult in humans. High-field neuroimaging at 7T is just emerging as a technique to assess the integrity of the human olfactory bulb. Optimizing this technique will require significant research, though there are promising results with T2prep BOLD fMRI for functional MRI of the bulb during olfactory tasks in humans (Miao et al. 2021).

#### Develop treatments for chemosensory impairment that are effective in older adults

The most consistent finding in the USA Smell and Taste Patient Survey was the poor efficacy in older adults of even the most commonly prescribed treatments for olfactory dysfunction (see Figs. 1 and 3). It has been well-validated that the prevalence of olfactory impairment in the population increases with age and is highest in older adults (Murphy et al. 2002; Hoffman et al. 2016). Jafari and Holbrook (2022) cite aging as the most frequent etiology of olfactory impairment. There is increased urgency in developing evidence-based treatments for olfactory dysfunction in older adults and including older adults in clinical trials for all novel treatment strategies.

The olfactory bulb and the central nervous system areas that process olfactory information are also areas that develop very early pathology in Parkinson’s disease (Braak et al. 2003) and Alzheimer’s disease (Braak and Braak 1995; Christen-Zaech et al. 2003; Wesson et al. 2010; Murphy 2019). Impaired performance on olfactory testing is seen in Parkinson’s disease (Ansari and Johnson 1975; Doty et al. 1988) and Alzheimer’s disease (Murphy et al. 2003; Wesson et al. 2010; Murphy 2019).

The wave of Parkinson’s disease after the 1918 Spanish flu as well as evidence that influenza is associated with a 70% higher risk of Parkinson’s disease 10 years later link insult to the olfactory bulb with Parkinson’s disease and Lewy Body Disease (Beauchamp et al. 2020; Cocoros et al. 2021; Kay 2022).

The fact that the elderly disproportionately develop severe COVID and long COVID raises the concern that olfactory impairment associated with COVID may increase the population's risk for Alzheimer’s disease. At the 2022 Alzheimer’s Association International Conference Gonzales-Aleman et al presented data from 766 Amerindian participants that demonstrated better prediction of cognitive impairment following COVID infection from severity of anosmia than from clinical status (Gonzales-Aleman et al. 2022). A study by Wang et al. (2022) using electronic health records of both inpatient and outpatient visits between 2/2020 and 5/2021, revealed a significant increase in new-onset AD within 360 days after initial COVID diagnosis. Data represented a cohort of 6,245,282 adults 65 and older with no prior diagnosis of AD from 68 healthcare organizations representing 28% of Americans from 50 states. Longer-term follow-up of patients, particularly those with long COVID, is urgent (Kay 2022). Recent research on more than 56,000 US Veterans demonstrated a 25% reduced risk of 10 long COVID symptoms, including cognitive impairment, in those who were treated with Paxlovid in the acute phase (Xie et al. 2023). Whether immediate treatment of COVID would decrease the prevalence of long-COVID-associated olfactory impairment and whether more effective treatments for olfactory dysfunction due to other etiologies would reduce the potential for long-term loss of chemosensory function or neurodegenerative disease are not known. Older adults with olfactory impairment are at increased risk for developing Alzheimer’s disease (Graves et al. 1999; Schubert et al. 2008; Wesson et al. 2010; Albers et al. 2015; Woodward et al. 2017; Murphy 2019; Wheeler and Murphy 2021). Long COVID compromises the integrity of brain areas that process both olfaction and memory (Douaud et al. 2022; Frank et al. 2024). We speculate that long COVID-associated olfactory impairment has the potential to drive a significant rise in AD. At present it is unknown whether olfactory impairment results from neurodegenerative disease or whether it hastens the development of neurodegenerative disease. It is essential to incorporate these and other questions related to COVID into the research agenda.

#### Medical provider education and training: Inform and strengthen research training, career development, and medical provider education in chemosensory disorders, assessment, and treatment strategies

Many survey patients commented about the lack of information about chemosensory disorders, assessment, and treatment strategies at the primary care level. The development of strategies to disseminate information about chemosensory disorders, assessment, and treatment strategies at the training, career development, and medical provider levels will be key to serving this patient population. Patient-centered outcome research indicates that there is a particular need not only for patient education about chemosensory testing but for medical provider education to increase its implementation. Engaging patients in partnerships with researchers and clinicians can provide enhanced education and training. Scientists and clinicians can help patients and patient advocacy groups by providing education and training on topics related to their research, such as disease mechanisms, treatment options, and clinical trial design. This can help chemosensory patient groups assist their constituents to better understand the scientific background for diagnosis and treatment and communicate patients’ needs and priorities more effectively to researchers and policymakers.

#### Ensure diversity in research, clinical trials, and in training of the next generation of chemosensory scientists and clinicians

Research on treatments for chemosensory disorders, including COVID-associated impairment, needs to reflect the diversity of our population. Ensuring that the next generation of chemosensory scientists and clinicians reflects this diversity may well facilitate that important goal. Efforts to achieve this have been made by the Association for Chemoreception Sciences in the AChemS Statement on suggestions to advance and strengthen racial equity, diversity, and inclusion in the biomedical research workforce and advance health disparities and health equity research. NIH training programs, particularly those funded by the NIDCD and the NIA, are mechanisms that can help foster these efforts. With the attention focused on chemosensory function and dysfunction by COVID, there is heightened potential to attract a diverse workforce into biomedical research into chemosensory impairment. Early encouragement at the undergraduate level through NIH diversity programs such as Minority Access to Research Careers (MARC), Advancing Diversity in Aging Research (ADAR), Minority Biomedical Research Support Program (MBRS), and research supplements to increase diversity in health-related research increases later participation in research careers. Earlier exposure can only enhance this process. Diversity fosters innovation and discovery and enhances research environments by increasing the participation of underserved or heath disparity populations in clinical studies. Diversity increases the likelihood that research outcomes that will inform effective prevention and treatment strategies for chemosensory impairment will benefit all. An NSF fact sheet on diversity programs is found at https://www.nsf.gov/news/factsheets/NSF_BP_FS_508.pdf. The NIH portal to funding diversity programs is https://extramural-diversity.nih.gov/

### Priorities for Research and Education

1. Develop partnerships with patient groups, clinicians, and basic scientists to facilitate patient-centered research, education, discovery, and therapeutics.
2. Develop chemosensory testing strategies that can be used for universal smell testing for population screening and by primary care providers and engage in high-level evidence-based studies to demonstrate utility to provide diagnostic value.
3. Use biomedical data science to investigate chemosensory disorders through electronic health records, epidemiological studies, and other large data sets.
4. Improve prevention, diagnosis, and treatment through investigation of the underlying etiologies of olfactory dysfunction.
5. Subject treatment strategies to randomized, clinical trials with control groups and patients across the lifespan.
6. 6 Develop a precision medicine approach to the prevention, diagnosis, and treatment of chemosensory disorders. Develop treatments for olfactory impairment that are effective in older adults.
7. 7 Build on lessons learned from COVID on the underlying mechanisms and interactions between immune response and regeneration.
8. 8 Facilitate basic research to enhance understanding of normal and disordered chemosensory function at the peripheral, central, clinical, and population levels.
9. 9 Develop and improve preclinical models to more closely resemble human disease to investigate underlying mechanisms of normal and disordered chemosensory function.
10. 10 Medical provider education and training: Inform and strengthen research training, career development, and medical provider education in chemosensory disorders. Ensure diversity in research, clinical trials, and in training the next generation of chemosensory scientists and clinicians.

## Supplementary Material

bjae020_suppl_Supplementary_Material

## Data Availability

The data underlying this article will be shared on reasonable request to the corresponding author.
