# Supplementary material for: Integrating the patient’s voice into the research agenda for treatment of chemosensory disorders
Source: Chem Senses. 2024 May 18;49:bjae020. doi: 10.1093/chemse/bjae020 (PMC11154145; doi:10.1093/chemse/bjae020)
Supplement: Supplementary file 1 [file bjae020_suppl_supplementary_material.docx]

PCORI "Shared Agenda for Clinical Research on Smell and Taste" Patient Survey

Start of Block: Screening Questions

Q68 You are being asked to participate in a survey that is led by the Monell Chemical Senses Center, Smell and Taste Association of North America (STANA), and Jefferson Health and funded by a grant from the Patient-Centered Outcomes Research Institute (PCORI). 

The survey is limited to residents of the United States of America. If you are a parent or caregiver, please complete the survey on behalf of your affected family member. 

 The survey should take about 15 minutes to complete. Upon completion, you can opt to enter and provide your contact information for a raffle for one of twenty-five $50 gift cards.

Your survey responses will be anonymous and no personally identifying information will be associated with the survey. There are no known risks or discomforts associated with answering these questions. Your participation is completely voluntary and if you choose not to participate, to discontinue participation in this study, or skip some questions there will be no penalty or loss of benefits, and this will not affect your future interactions with Monell, STANA or Jefferson Health. 

The purpose of this research survey is to understand the impact of smell or taste dysfunction from COVID-19 and other causes on a variety of everyday experiences and to gain a better understanding of any barriers in diagnosis or treatment for smell and taste loss. Monell, STANA and Jefferson Health have partnered to develop a prioritized research agenda that creates a framework for future patient-centered research, including updating a 2018 White Paper outlining research needed to advance treatments. 

Your answers will be kept confidential. The data collected from this survey will be used for research purposes only, will be analyzed and stored by the study team at Monell, STANA and Jefferson, and may be used in the future to answer other research questions.

If you have any questions, concerns, or complaints regarding your participation in this survey, please contact: the Principal Investigator, Dr. Pamela Dalton, at 267-519-4810 or dalton@monell.org, or the Co-Investigator, Katie Boateng, at info@stana.org. If you would like to speak with someone other than the study sponsors, you may contact the Office of Regulatory Affairs with any question, concerns, or complaints at the University of Pennsylvania by calling 215-573-2540.

| Page Break |  |
| --- | --- |

Q82 Please state whether you consent to participate in this study:  

- Yes, I consent to participate in this study  (1) 

- No, I do not consent to participate in this study  (2) 

Skip To: End of Block If Please state whether you consent to participate in this study:  = No, I do not consent to participate in this study

| Page Break |  |
| --- | --- |

Q1 Relationship to smell and/or taste disorder 

- Patient  (1) 

- Family or caregiver of a patient (not a parent)  (2) 

- Parent of a patient  (3) 

- None of the above  (4) 

Skip To: End of Block If Relationship to smell and/or taste disorder  = None of the above

| Page Break |  |
| --- | --- |

Q66 Country of Residence

- United States of America  (1) 

- Other  (2) 

Skip To: End of Block If Country of Residence = Other

Q4 Throughout this survey, you are going to be asked to rate your senses of taste and smell. 

Taste is defined as the ability to perceive chemicals on the tongue as sweet, salty, sour, bitter, or umami/savory (for example cooked mushrooms or soy sauce). 

Smell is defined as the ability to  perceive odors or scents. 

End of Block: Screening Questions

Start of Block: Demographic Information

Q6 Gender

- Male  (1) 

- Female  (2) 

- Non-binary / third gender  (3) 

- Prefer not to say  (4) 

- Other (fill in the blank below)  (5) ________________________________________________

Q7 Age

- Under 18  (1) 

- 18 - 24  (2) 

- 25 - 39  (3) 

- 40-60  (4) 

- 60 or older  (5) 

Q8 Do you consider yourself:

- White  (1) 

- Black or African American  (2) 

- American Indian or Alaska Native  (3) 

- Asian  (4) 

- Native Hawaiian or Pacific Islander  (5) 

- Prefer not to answer  (6) 

- Other (fill in the blank below)  (7) ________________________________________________

Q9 Are you of Spanish or Hispanic origin, such as Latin American, Mexican, Puerto Rican, Cuban, or Dominican? 

- Yes, of Hispanic origin  (1) 

- No, not of Hispanic origin  (2) 

- Prefer not to answer  (3) 

state In which state do you currently reside?

▼ Alabama (1) ... I do not reside in the United States (53)

|  |
| --- |

Q78 What is your zip code? 

________________________________________________________________

Q17 What type of health insurance do you have?

- I do not have any health insurance  (1) 

- Individual health insurance policy (purchased directly from a health insurer or through a health insurance exchange)  (2) 

- Employer or other group health insurance  (3) 

- Military/TRICARE  (4) 

- Medicare  (5) 

- Medicaid  (6) 

- Other (fill in the blank below)  (7) ________________________________________________

Q18 Do you see a healthcare provider for preventative visits or regular check-ups? 

- Yes  (1) 

- No  (2) 

Skip To: Q19 If Do you see a healthcare provider for preventative visits or regular check-ups?  = Yes

Q19 What type of healthcare provider do you see for preventative visits or regular check-ups? 

- Primary Care Clinician (MD, DO, NP, PA, etc.)  (1) 

- Otolaryngologist (Ear Nose Throat - ENT) Specialist  (7) 

- Nurse practitioner  (8) 

- Neurologist  (9) 

- Chiropractor  (10) 

- Dentist  (3) 

- Acupuncturist  (11) 

- Taste and Smell Clinic  (12) 

- Allergist  (13) 

- Oncologist  (14) 

- OB/Gynecologist  (2) 

- Eye doctor (ophthalmologist) or optometrist  (4) 

- Other (fill in the blank below)  (5) ________________________________________________

End of Block: Demographic Information

Start of Block: Disease Experience/Diagnosis

Q20 Which of the following have you ever experienced? Check all that apply. 

- Complete smell loss (anosmia)  (4) 

- Reduced smell loss or partial loss of smell (hyposmia)  (5) 

- Distorted smells (parosmia)  (6) 

- Phantom smells (phantosmia)  (7) 

- Complete taste loss (ageusia)  (1) 

- Distorted taste (dysgeusia/parageusia)  (3) 

- Reduced taste loss or partial loss of taste (hypogeusia)  (2) 

- None  (8) 

Q31 Is your smell/taste dysfunction due to Covid-19? 

- Yes  (1) 

- No  (2) 

- I don't know  (3) 

Q77 What would you like your healthcare provider to know about your condition? 

________________________________________________________________

________________________________________________________________

________________________________________________________________

________________________________________________________________

________________________________________________________________

Q22 Is your smell/taste dysfunction officially documented in your medical record? 

- Yes  (1) 

- No  (2) 

- I don't know  (3) 

Display This Question:

If Is your smell/taste dysfunction officially documented in your medical record?  = Yes

Q23 Which of the following is officially documented in your medical record? 

- Complete smell loss (anosmia)  (4) 

- Reduced smell loss or partial loss of smell (hyposmia)  (5) 

- Distorted smells (parosmia)  (6) 

- Phantom smells (phantosmia)  (7) 

- Complete taste loss (ageusia)  (1) 

- Distorted taste (dysgeusia/parageusia)  (3) 

- Reduced taste loss or partial loss of taste (hypogeusia)  (2) 

- I don't know  (8) 

Display This Question:

If Which of the following is officially documented in your medical record?  = Distorted smells (parosmia)

Q33 What do you think caused your parosmia? 

- Cold/flu  (1) 

- Allergies  (2) 

- Trauma to the nose or face  (3) 

- Stroke or other neurologic event  (4) 

- Migraine  (5) 

- Virus (not Covid-19)  (6) 

- Covid-19 (virus)  (8) 

- Other (fill in the blank below)  (7) ________________________________________________

Display This Question:

If Which of the following is officially documented in your medical record?  = Distorted smells (parosmia)

Q36 What types of things trigger your parosmia? 

________________________________________________________________

Display This Question:

If Which of the following have you ever experienced? Check all that apply.  = Complete smell loss (anosmia)

Q74 What do you think caused your anosmia?

- Traumatic brain injury (TBI)  (1) 

- Congenital  (2) 

- Viral (not related to Covid-19)  (3) 

- Viral (Covid-19)  (4) 

- Aging  (6) 

- Idiopathic (cause is unknown)  (7) 

- Other (fill in the blank below)  (5) ________________________________________________

Display This Question:

If Is your smell/taste dysfunction officially documented in your medical record?  = No

Q25 Diagnosis status

- I do not have an official diagnosis but I am certain I have one of these conditions  (1) 

- I do not have an official diagnosis and I am unsure whether I have one of these conditions  (2) 

| Page Break |  |
| --- | --- |

|  |
| --- |

 
To the best of your knowledge, when did you first **experience** your symptoms of smell/taste dysfunction? 
Select a Date (feel free to approximate): 
 

|  | Month | Year |
| --- | --- | --- |
|  |  |  |
| Please Select: (1) | ▼ January (1 ... December (12) | ▼ 1900 (1 ... 2049 (150) |

Display This Question:

If Is your smell/taste dysfunction officially documented in your medical record?  = Yes

|  |
| --- |

Q27 
To the best of your knowledge, when were you first **diagnosed** with your symptoms of smell/taste dysfunction? If not applicable (for example, you do not have an official diagnosis) please leave blank. 
Select a Date (feel free to approximate):

 

|  | Month | Year |
| --- | --- | --- |
|  |  |  |
| Please Select: (1) | ▼ January (1 ... December (12) | ▼ 1900 (1 ... 2049 (150) |

|  |
| --- |

Q4 
To the best of your knowledge, when did your smell/taste dysfunction **end**? If not applicable (for example, you're still experiencing it) please leave blank. 
Select a Date (feel free to approximate): 

 

|  | Month | Year |
| --- | --- | --- |
|  |  |  |
| Please Select: (1) | ▼ January (1 ... December (12) | ▼ 1900 (1 ... 2049 (150) |

| Page Break |  |
| --- | --- |

Q29 How would you have rated your sense of smell prior to the onset of your symptoms? (Smell is defined as the ability to perceive odors or scents.) 

- Extremely bad  (1) 

- Somewhat bad  (2) 

- Neither good nor bad  (3) 

- Somewhat good  (4) 

- Extremely good  (5) 

Q30 How would you have rated your sense of taste prior to the onset of your symptoms? (Taste is defined as the ability to perceive chemicals on the tongue as salty, sweet, sour, bitter, or umami/savory [example: cooked mushrooms or soy sauce].)

- Extremely bad  (1) 

- Somewhat bad  (2) 

- Neither good nor bad  (3) 

- Somewhat good  (4) 

- Extremely good  (5) 

Q37 Once you realized you were experiencing smell/taste dysfunction, which of the following did you do? 

- Visit a healthcare provider  (1) 

- Look for information online  (2) 

- Talk to friends and family  (3) 

- Other (fill in the blank below)  (4) ________________________________________________

Display This Question:

If Once you realized you were experiencing smell/taste dysfunction, which of the following did you do?  = Look for information online

Q38 If you sought out information on social media, which of the following did you seek out? Check all that apply. 

- Facebook  (1) 

- Twitter  (2) 

- LinkedIn  (3) 

- TikTok  (4) 

- Instagram  (6) 

- Other (fill in the blank below)  (5) ________________________________________________

Display This Question:

If Once you realized you were experiencing smell/taste dysfunction, which of the following did you do?  = Look for information online

Q39 If you looked online for information, which of the following places did you use? Select all that apply. 

- Academic websites (Mayo, Hopkins, etc.)  (1) 

- General search (Google, Bing, Yahoo, etc.)  (2) 

- Other (fill in the blank below)  (3) ________________________________________________

End of Block: Disease Experience/Diagnosis

Start of Block: Medical Provider

Q40 Did you see a healthcare provider for your symptoms? 

- No  (1) 

- Yes  (2) 

Display This Question:

If Did you see a healthcare provider for your symptoms?  = No

Q41 Why not?

________________________________________________________________

________________________________________________________________

________________________________________________________________

________________________________________________________________

________________________________________________________________

Display This Question:

If Did you see a healthcare provider for your symptoms?  = Yes

Q42 What type of healthcare provider did you visit? Check all that apply. 

- Family practitioner  (1) 

- Otolaryngologist (Ear Nose Throat - ENT) Specialist  (2) 

- Nurse practitioner  (3) 

- Neurologist  (4) 

- Chiropractor  (5) 

- Dentist  (6) 

- Acupuncturist  (7) 

- Taste and Smell Clinic  (8) 

- Allergist  (9) 

- Oncologist  (10) 

- Other (fill in the blank below)  (11) ________________________________________________

End of Block: Medical Provider

Start of Block: Follow up Questions to Q42

Q56 Did the ${lm://Field/1} do any of the following? Check all that apply. 

- Provide written materials  (1) 

- Provide a referral to another specialist  (2) 

- Provide social support  (3) 

- None of the above  (5) 

- Other (fill in the blank below)  (4) ________________________________________________

Q57 How knowledgeable was the ${lm://Field/1} about smell/taste dysfunction? 

- Not knowledgeable at all  (1) 

- Slightly knowledgeable  (2) 

- Moderately knowledgeable  (3) 

- Very knowledgeable  (4) 

- Extremely knowledgeable  (5) 

Q58 How supported (listened to and believed) by the ${lm://Field/1} did you feel during your visit? 

- Very supported  (1) 

- Supported  (2) 

- Somewhat supported  (3) 

- Not at all supported  (4) 

Q59 How supported (listened to and believed) by the ${lm://Field/1} did you feel after your visit? 

- Very supported  (1) 

- Supported  (2) 

- Somewhat supported  (3) 

- Not at all supported  (4) 

Q60 What was your confidence level that the ${lm://Field/1} could help you manage or treat your symptoms? 

- Very confident  (1) 

- Confident  (2) 

- Somewhat confident  (3) 

- Not at all confident  (4) 

End of Block: Follow up Questions to Q42

Start of Block: Diagnosis

Display This Question:

If Did you see a healthcare provider for your symptoms?  = Yes

Q43 If you had a difficult time getting a diagnosis, are there any specific reasons you think played a role in that difficulty? Check all that apply. 

- I do not feel like I was listened to and/or believed  (1) 

- Lack of health insurance  (2) 

- Lack of provider knowledge about diagnostic testing  (4) 

- The cost of testing was prohibitive  (5) 

- Difficulty getting access to a specialist to get a diagnosis  (6) 

- My symptoms were attributed to another disease that I already had a diagnosis for  (7) 

- I did not have a hard time getting diagnosed  (8) 

- Other (fill in the blank below)  (9) ________________________________________________

Display This Question:

If Did you see a healthcare provider for your symptoms?  = Yes

Q44 What tests or diagnostic procedures did you undergo? Check all that apply. 

- Smell test (UPSIT, Sniffin' Sticks, etc.)  (1) 

- Taste test  (2) 

- MRI  (3) 

- CT  (4) 

- Nasal endoscopy  (5) 

- Questionnaire (Example: SNOT-22)  (6) 

- Medical history  (7) 

- Other (fill in the blank below)  (8) ________________________________________________

- None of the above  (9) 

Display This Question:

If Did you see a healthcare provider for your symptoms?  = Yes

Q45 How many healthcare providers did you see for the problem before you got a diagnosis? 

- 1  (1) 

- 2  (2) 

- 3  (3) 

- more than 3  (6) 

- I do not have a diagnosis  (7) 

- Other (fill in the blank below)  (8) ________________________________________________

Display This Question:

If Did you see a healthcare provider for your symptoms?  = Yes

Q46 What treatment options did your healthcare provider recommend? Check all that apply. 

- Nasal steroids  (1) 

- Oral steroids  (2) 

- Zinc  (3) 

- Nasal rinse  (4) 

- Smell training  (5) 

- Alpha lipoic acid  (6) 

- Theophylline  (7) 

- Omega-3  (8) 

- PRP (platelet rich plasma)  (9) 

- Participating in a clinical trial  (10) 

- Other (fill in the blank below)  (11) ________________________________________________

- None of the above  (12) 

End of Block: Diagnosis

Start of Block: Follow up to Q46 : What Treament 

Q62 Do you/did you face any of the following barriers or difficulties in accessing your selected treatment: (${lm://Field/1})? 

- Cost  (1) 

- Insurance coverage  (2) 

- Physical proximity to appointments for treatment  (3) 

- Amount of time the treatment takes  (4) 

- Side effects from the treatment  (5) 

- Other (fill in the blank below)  (6) ________________________________________________

- I do not/did not face any barriers or difficulties  (7) 

Q61 If (${lm://Field/1}) was used, please rate how effective it has been. 

- Not effective at all  (1) 

- Slightly effective  (2) 

- Moderately effective  (3) 

- Very effective  (4) 

- Extremely effective  (5) 

End of Block: Follow up to Q46 : What Treament 

Start of Block: Condition's Impact on Quality of Life

Q37 To what degree (if any) has your smell/taste dysfunction affected the following lifestyle functions for you?

|  | Not at all (1) | Somewhat (2) | Mildly or slightly (3) | Moderately (4) | Very (5) | Extremely (6) |
| --- | --- | --- | --- | --- | --- | --- |
| Nutrition (diet, weight loss, weight gain) (1) |  |  |  |  |  |  |
| Eating habits (quantity, preferences, other changes) (2) |  |  |  |  |  |  |
| Cooking ( level of enjoyment, frequency, ability) (3) |  |  |  |  |  |  |
| Food choices (color, texture, temperatures) (4) |  |  |  |  |  |  |
| Appetite (5) |  |  |  |  |  |  |
| Relationship and bonding with others (significant others, family members, children, etc. (6) |  |  |  |  |  |  |
| How much you enjoy dining out with others (7) |  |  |  |  |  |  |
| Work (8) |  |  |  |  |  |  |
| Mental health (depression, anxiety, grief) (9) |  |  |  |  |  |  |
| Memory (10) |  |  |  |  |  |  |
| Pleasure/Joy (11) |  |  |  |  |  |  |
| Safety (12) |  |  |  |  |  |  |
| Sex and intimacy (13) |  |  |  |  |  |  |
| Personal hygiene habits (14) |  |  |  |  |  |  |
| How much your worry/focus on hygiene (15) |  |  |  |  |  |  |
| Other (fill in the blank below) (16) |  |  |  |  |  |  |

Q40 Is there anything you wish others understood about the impact of smell/taste dysfunction on your quality of life? 

- Yes  (1) 

- No  (2) 

Display This Question:

If Is there anything you wish others understood about the impact of smell/taste dysfunction on your... = Yes

Q41 What do you wish others understood about the impact of smell/taste dysfunction on your quality of life? 

________________________________________________________________

________________________________________________________________

________________________________________________________________

________________________________________________________________

________________________________________________________________

| Page Break |  |
| --- | --- |

Q42 Have you specifically sought treatment for your mental health in dealing with your smell/taste dysfunction?

- Yes  (1) 

- No  (2) 

Q43 Do you think your smell/taste dysfunction is a disability? 

- Yes  (1) 

- No  (2) 

Display This Question:

If Do you think your smell/taste dysfunction is a disability?  = Yes

Q44 Please expand on why you think your smell/taste dysfunction is a disability. 

________________________________________________________________

________________________________________________________________

________________________________________________________________

________________________________________________________________

________________________________________________________________

Display This Question:

If Do you think your smell/taste dysfunction is a disability?  = No

Q45 Please expand on why you think your smell/taste dysfunction is **not** a disability.

________________________________________________________________

________________________________________________________________

________________________________________________________________

________________________________________________________________

________________________________________________________________

| Page Break |  |
| --- | --- |

Q46 Of all the possibilities for support for people with smell/taste dysfunction, please rate the following items on level of importance to you.

|  | Not at all important (1) | Slightly important (2) | Moderately important (3) | Very important (4) | Most Important (5) |
| --- | --- | --- | --- | --- | --- |
| More public awareness about the condition (1) |  |  |  |  |  |
| More medical community knowledge about the condition (2) |  |  |  |  |  |
| Effective treatments for the condition (3) |  |  |  |  |  |
| Better diagnostic tools for the condition (4) |  |  |  |  |  |
| Supportive devices (electronic noses, gas detection, etc.) (5) |  |  |  |  |  |
| Lifestyle strategies for compensating (6) |  |  |  |  |  |
| Mental health support (10) |  |  |  |  |  |
| Other (fill in the blank below) (7) |  |  |  |  |  |
| Other (fill in the blank below) (8) |  |  |  |  |  |
| Other (fill in the blank below) (9) |  |  |  |  |  |

End of Block: Condition's Impact on Quality of Life

Start of Block: Information/Systems for Support 

Q47 What are your primary sources of information about smell/taste dysfunction? Check all that apply. 

- The provider I see for my smell and/or taste loss or distortion  (1) 

- Friends/Family  (2) 

- Other people with my disorder  (3) 

- Social media sites (Twitter, Facebook, TikTok, Instagram, etc.)  (4) 

- Search engines like Google  (5) 

- Academic/scientific organization websites  (6) 

- Patient advocacy organizations  (7) 

- Magazines, books, or newsletters  (8) 

- Television or radio  (9) 

- In-person events or educational classes  (10) 

- I do not seek any information  (11) 

- Other (fill in the blank below)  (12) ________________________________________________

Q48 What are your primary sources of support for dealing with or coping with your smell/taste dysfunction? Check all that apply. 

- The provider I see for my smell and/or taste loss or distortion  (1) 

- Friends/Family  (2) 

- Other people with my disorder  (3) 

- Social media sites (Twitter, Facebook, TikTok, Instagram, etc.)  (4) 

- Search engines like Google  (5) 

- Academic/scientific organization websites  (6) 

- Patient advocacy organizations  (7) 

- Magazines, books, or newsletters  (8) 

- Television or radio  (9) 

- In-person events or educational classes  (10) 

- I do not seek any support  (11) 

- Other (fill in the blank below)  (12) ________________________________________________

End of Block: Information/Systems for Support 

Start of Block: Looking Ahead

|  |
| --- |

Q67 If a new treatment were to be developed for smell/taste dysfunction, what are the top three factors that would impact whether or not you would try it? 

- Effectiveness of the drug or treatment for treating the most troublesome symptoms  (1) 

- Cost or insurance barriers  (2) 

- Method of administration (injection, infusion, oral, topical)  (3) 

- Required frequency of treatments  (4) 

- Required frequency administering doses  (5) 

- Psychological effect (fear, nervousness) of the therapy  (6) 

- Common but not serious side effects (headache, nausea, etc.)  (7) 

- Uncommon but serious side effects (risk of cancer, anaphylaxis, etc.)  (8) 

- Travel distance to clinic/provider to receive the therapy  (9) 

- Duration of treatment (days, weeks, months to complete)  (11) 

- Other (fill in the blank below)  (10) ________________________________________________

Q50 Would you be willing to participate in research for smell/taste dysfunction? 

- Yes  (1) 

- No  (2) 

Display This Question:

If Would you be willing to participate in research for smell/taste dysfunction?  = Yes

Q51 Of the following types of research, rate how likely you would be to participate

|  | Extremely unlikely (1) | Somewhat unlikely (2) | Neither likely nor unlikely (3) | Somewhat likely (4) | Extremely likely (5) |
| --- | --- | --- | --- | --- | --- |
| Clinical trial for a new pharmaceutical treatment (1) |  |  |  |  |  |
| Research project for a surgical treatment (2) |  |  |  |  |  |
| Research project for a non-pharmaceutical or non-surgical treatment (example: a study about the effectiveness of smell training) (3) |  |  |  |  |  |
| Research focused on causes or risk factors for smell/taste disorders or mechanisms for smell and taste regeneration (example: genetic testing to identify genes involved in smell loss or regeneration) (5) |  |  |  |  |  |

Display This Question:

If Would you be willing to participate in research for smell/taste dysfunction?  = No

Q52 Why not?

________________________________________________________________

Q53 In your opinion, what is the most effective way to inform patients about advances in research and treatments for smell/taste dysfunction? 

________________________________________________________________

________________________________________________________________

________________________________________________________________

________________________________________________________________

________________________________________________________________

Q54 When you think about the future, do any of the following items concern you? Check all that apply. 

- Your sense of smell and/or taste will only recover partially  (1) 

- Your sense of smell and/or taste will not recover at all  (2) 

- Your sense of smell and/or taste may be lost or distorted again  (3) 

- None of the above  (5) 

- Other (fill in the blank below)  (4) ________________________________________________

Q58 Do you feel like you have been treated differently in any of the following environments? Check all that apply. 

- Workplace  (1) 

- Family  (2) 

- Social setting  (3) 

- Other (fill in the blank below)  (4) ________________________________________________

- I do not feel like I've been treated differently  (5) 

| Page Break |  |
| --- | --- |

Q55 How do people react when they hear about your smell/taste dysfunction? 

________________________________________________________________

________________________________________________________________

________________________________________________________________

________________________________________________________________

________________________________________________________________

Q57 What do you think people find most difficult to understand about your smell/taste dysfunction?

________________________________________________________________

________________________________________________________________

________________________________________________________________

________________________________________________________________

________________________________________________________________

Q21 Are there any other aspects of your smell/taste dysfunction you would like to share with us? 

________________________________________________________________

________________________________________________________________

________________________________________________________________

________________________________________________________________

________________________________________________________________

End of Block: Looking Ahead
